# Composite Alloplastic Biomaterial vs. Autologous Platelet-Rich Fibrin in Ridge Preservation

**DOI:** 10.3390/jcm8020223

**Published:** 2019-02-09

**Authors:** Gerardo Mendoza-Azpur, Allinson Olaechea, Miguel Padial-Molina, Lourdes Gutiérrez-Garrido, Francisco O’Valle, Francisco Mesa, Pablo Galindo-Moreno

**Affiliations:** 1 Department of Periodontology, School of Dentistry, Cientifica del Sur University, Lima 15067, Peru; drgerardoodonto@yahoo.com (G.M.-A.); allyolaechea@gmail.com (A.O.); 2 Department of Oral Surgery and Implant Dentistry, School of Dentistry, University of Granada, 18071 Granada, Spain; lourdesgutierrezgarrido@gmail.com (L.G.-G.); pgalindo@ugr.es (P.G.-M.); 3 Department of Pathology & Institute of Biopathology and Regenerative Medicine (IBIMER, CIBM), University of Granada, 18071 Granada, Spain; fovalle@ugr.es; 4 Department of Periodontology, School of Dentistry, University of Granada, 18071 Granada, Spain; fmesa@ugr.es

**Keywords:** beta triphasic calcium phosphate (β-TCP), platelet-rich fibrin (PRF-L), ridge augmentation, post-extraction socket

## Abstract

Aim: The aim of this study was to examine the clinical and histological differences of using a combination of alloplastic beta triphasic calcium phosphate (β-TCP) and a cross-linked collagen membrane versus autologous platelet-rich fibrin (PRF-L) in ridge preservation after dental extraction. Material and methods: Fifty-one patients were included in this observational case-series study. Dental extractions were performed, after which 25 patients were grafted with β-TCP and 26 with PRF-L. After four months of healing, clinical, radiological, histomorphometric and histological evaluations were performed. Results: A significantly higher percentage of mineralized tissue was observed in samples from the PRF-L grafted areas. Cellularity was higher in PRF-L grafted areas (osteocytes in newly formed bone per mm^2^ = 123.25 (5.12) vs. 84.02 (26.53) for PRF-L and β-TCP, respectively, *p* = 0.01). However, sockets grafted with PRF-L showed a higher reduction in the bucco-lingual dimension after four months of healing (2.19 (0.80) vs. 1.16 (0.55) mm, *p* < 0.001), as well as a higher alteration in the final position of the mid muco-gingival junction (1.73 (1.34) vs. 0.88 (0.88) mm, *p* < 0.01). Conclusion: PRF-L concentrate accelerates wound healing in post-extraction sockets in terms of new mineralized tissue component. However, the use of β-TCP biomaterial appears to be superior to maintain bucco-lingual volume and the final position of the muco-gingival junction.

## 1. Introduction

A sufficient amount of functional and healthy bone is a very important factor for getting and maintaining osseointegration. Crest height and width are reduced to about 40%–60% of their original after a tooth is extracted, mainly within the first year following extraction [1,2,3]. Consequently, soft tissue complications and long-term success of implants placed in compromised sites can be expected [4,5,6]. Thus, socket filling at the time of extraction is recommended to prevent such complications [7,8].

Filling materials are numerous, with distinct properties and advantages and disadvantages in terms of osteogenesis, osteoconduction, osteoinduction, resorption rates and handling, among others. Allografts [9] and xenografts [10] are the most commonly used and studied. Another group includes alloplastic materials, which are usually made of calcium phosphate with either hydroxyapatite, tricalcium phosphate (TCP), or a combination known as biphasic calcium phosphates [11]. Tricalcium phosphate exhibits better biodegradation properties, being completely replaced by newly formed bone [11]. Tricalcium phosphates under the allotropic forms (α and β) are resorbable, nontoxic, and do not promote irritation, inflammatory, or immune responses, and consequently foreign body reaction [12]. During the degradation process, TCP releases calcium and phosphate ions in the local tissue, promoting bone formation by stimulation of osteoblastic cells [13]. According to its high water solubility, TCP is dissolved in tissue fluids and absorbed by osteoclasts in vivo [14]. This process happens slowly, which is generally recognized to be ideal in a bony biomaterial [15]. It also provides a three-dimensional matrix for new bone deposition, against the pressure of tissue shrinkage, showing also a substantial physical strength [16]. Thus, it has been used in a number of clinical applications, from ridge preservation to sinus floor elevation [16].

On the other hand, platelet-rich fibrin (PRF) has been regarded as an inexpensive and low morbidity autograft [17]. It is prepared by centrifugation of whole blood drawn into a tube without anticoagulant. Platelet-rich fibrin is considered a rich source of cytokines and growth factors (particularly platelet-derived growth factor—PDGF-AB, transforming growth factor—TGF)-β, and vascular endothelial growth factor—VEGF), for which it has been recognized as a good healing inductor biomaterial [17]. It consists of a fibrin matrix that incorporates leukocytes and platelets [18]. This blood concentrate leads to more-efficient cell migration, proliferation, and angiogenesis [19]; improves immune control and recruitment of circulating stem cells; and enhances wound protection through the promotion of an epithelial cover [20]. PRF is considered to benefit soft tissue healing and bone regeneration [21,22]. Thus, it has been claimed as suitable autogenous material for socket preservation and ridge preservation [23].

The aim of this study was to determine the clinical and histological differences after using a combination of alloplastic triphasic calcium phosphate and a cross-linked collagen membrane versus autologous platelet-rich fibrin in ridge preservation after dental extraction.

## 2. Materials and Methods

This observational case-series study was conducted following the principles outlined in the Declaration of Helsinki and internationally consented ethics in clinical research [24]. A quality assessment was carried out based on the STrengthening the Reporting of OBservational studies in Epidemiology (STROBE) checklist [25]. The study protocol was approved by the Ethics on Research Committee of the School of Dentistry, Universidad Científica del Sur, Lima, Peru with registration number 00024, which is where clinical activities were performed. All patients received detailed oral and written information about the study, including the risks, benefits and alternative therapies, and signed an informed consent form before any study procedures.

### 2.1. Sample Size Calculation and Experimental Groups

The primary outcome variable was vital bone percentage. Assuming that differences in the measurement would not exceed 7%, the sample size was calculated to be 22 subjects per treatment group. This would provide 80% power and 5% 2-sided type 1 error. A total of 44 patients would be needed; the total number of patients was set to at least 50 to deal with potential withdrawals. Prior to surgery, patients were alternatively assigned to each group: beta triphasic calcium phosphate (0.5–1.0 mm particle size) (Osteon II, Genoss, Seoul, Korea) and a cross-linked collagen membrane group (Genoss, Seoul, Korea) (β-TCP group); and the autologous platelet-rich fibrin group (PRF-L group).

### 2.2. Patient Selection

Inclusion criteria were set to include patients aged >18, in good general health, in need of an uni-radicular tooth extraction (hopeless incisors or canines for periodontal, traumatic, or caries reasons with no peri-radicular radiolucent image), and expressing an interest in replacement with dental implants. Cases with a thin or partially missing labial plate (<50%) were included in the study. Patients were excluded if they had poor oral hygiene, were subject to irradiation in the head and neck area, were immunosuppressed or immunocompromised, were treated or were under treatment with intravenous aminobisphosphonates, had uncontrolled diabetes, were pregnant, were lactating or menopausal women, were substance abusers, had psychiatric problems, were smokers or user of electronic cigarettes, or presented any other contraindication for oral surgery.

### 2.3. PRF Management

Those in the PRF-L group were subjected to intravenous blood collection to obtain PRF-L clots according to a previously described protocol [20]. For each patient, one 10-mL vial without anticoagulant was collected and immediately centrifuged at 3000 revolutions per minute for 10 min. The fibrin clot formed in the middle part of the tube and was separated from the lower part of the centrifuged blood and spread on a sterile gauze to be used in the socket.

### 2.4. Surgical Procedures and Intrasurgical Measurements

To optimize the clinical measurements, a template was used as a reference to reproduce the initial position at the reentry procedure. Before any intervention was done, patients were asked to rinse for 30 s with an antiseptic mouthwash containing 0.2% chlorhexidine. Following this, the mucogingival junction (MGJ) at the mesial, buccal and distal positions of the study site were taken as the distance from the acrylic template. The tooth extraction was performed under local anesthesia (2% xylocaine with 1:100,000 epinephrine). An intrasulcular incision was extended along the study tooth to the neighboring teeth by elevation of buccal and lingual full-thickness flaps that did not extend beyond the MGJ. Tooth extraction was then carefully performed by periotome and the appropriate dental forceps to minimize surgical trauma on the surrounding hard tissue and the socket walls. Then, the postoperative bucco-lingual/palatal width (BLW) of the alveolar ridge at the midpoint of the alveolar crest, and the height of the alveolar bone crest (ABC) at three sites (mesial, buccal, and distal) with a customized acrylic template were recorded.

At this point, the socket was filled with either PRF-L or β-TCP, according to the assigned group. Those sockets in the β-TCP group were covered by a collagen membrane to prevent the biomaterial from getting out of the socket. In both cases, simple X interrupted sutures were used to reposition the flap over the augmented area by means of polyglycolic acid resorbable 5/0 sutures (Glicosorb, Tagum, Lima, Peru).

Oral and written post-operative instructions were given to the patients, and appropriate antibiotics (amoxicilin 750 mg/8 h for 7 days) and NSAIDs (ibuprofen 400 mg/8 h for 3 days) were prescribed. Sutures were removed 10 days post-surgery, and patients were re-evaluated at regular 1-month intervals during the 4 months of healing.

### 2.5. Surgical Reentry for Implant Placement

Patients were followed-up after 10 days, 1, 2 and 3 months. After 4 months, clinical MGJ before, and BLW and ABC after the flap release measurements were recorded again. Bone tissue biopsies of the grafted area were obtained using a 3 mm in/4-mm out trephine (Dentium Co., Ltd., Seoul, Korea) along the long axis of the treated site prior to implant placement and beyond the original native bone. The collected cores were immediately fixed in formalin 10% for 48 h at 4 °C and then transferred to ethanol 70% at 4 °C. Tapered dental implants (SuperLine, Dentium Co., Ltd., Seoul, Korea) were placed in each prepared site. Primary stability was achieved in all cases.

### 2.6. Histologic and Histomorphometric Evaluation

Fixed samples were transported to the Center for Biomedical Research at the University of Granada (CIBM-UGR) for evaluation. They were decalcified with 10% ethylene diaminetetraacetic acid (EDTA, Sigma-Aldrich Co., LLC, St. Louis, MO, USA) for 4 weeks. Following this, they were embedded in paraffin blocks and sectioned along the central axis of the biopsies. Sections were dewaxed, rehydrated and stained with conventional hematoxilin-eosin and Masson trichrome protocol. A millimeter scale in the eyepiece of a microscope with 40× objective was used to count the osteoblasts and osteocytes cells per mm². The results were expressed as number of cells per mm^2^.

Bone histomorphometry was performed semi-automatically on Masson trichrome-stained sections. Ten random images per sample, captured with a 10x objective, were evaluated using the ImageJ software (NIH, Bethesda, MA, USA). Mineralized and non-mineralized tissue were calculated. The results were expressed as a percentage.

### 2.7. Statistical Analysis

All data are presented as a mean (standard deviation). Statistical analysis was performed using a Student’s *t*-test for all measurements, either paired (before and after, MGJ, BLW, and ABC) or independent (histologic and histomorphometric data). When data were not normally distributed, a Mann–Whitney U test was performed. Statistical significance was set on a *p*-value of 0.05. SPSS 21.0 (IBM, Armonk, NY, USA) was used for the analyses.

## 3. Results

Table 1 summarizes the demographic data of the included patients (both total and per group).

**Figure 1:**
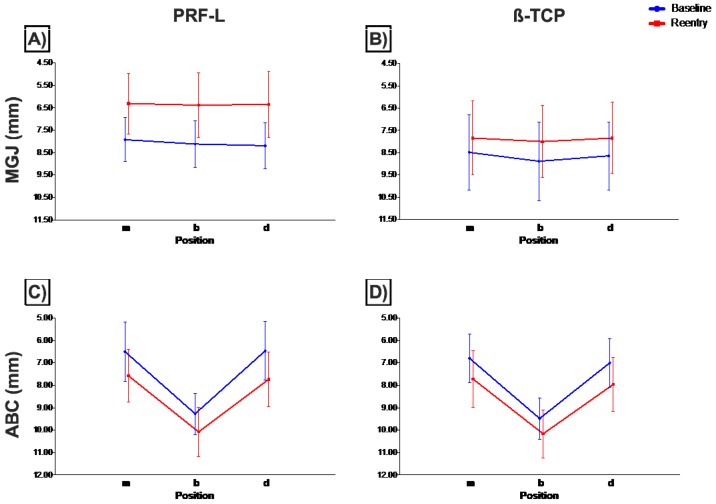
Mucogingival junction (**A**,**B**) and alveolar bone crest (**C**,**D**) measurements (mean and standard deviation) at baseline (blue) and reentry (red) at the three different positions for PRF-L (**A**–**C**) and β-TCP (**B**,**D**) groups. Definitions: m, mesial; b, buccal; d, distal; ABC, alveolar bone crest; MGJ, mucogingival junction; PRF-L, platelet-rich fibrin group; β-TCP, beta tricalcium phosphate group.

Analysis of clinical data shows several significant differences. MGJ changed from baseline to reentry in all positions within each group separately (*p* < 0.01, paired Student’s *t*-test) (Figure 1A,B). Comparison between PRF-L and β-TCP showed significant differences at reentry in all positions (*p* < 0.01, independent Student’s *t*-test). Notably, the analysis of the difference between each time point shows that for the β-TCP group, the MGJ moved coronally significantly less at the three positions (mesial, buccal, and distal) (*p* < 0.01, independent Student’s *t*-test).

In contrast, the analysis of the alveolar bone crest (ABC) showed no significant differences between groups at any time point or position (Figure 1C,D). Differences between baseline and reentry within each group were statistically significant at either of the three sites (mesial, buccal, and distal) (*p* < 0.01, paired Student’s *t*-test).

**Figure 2:**
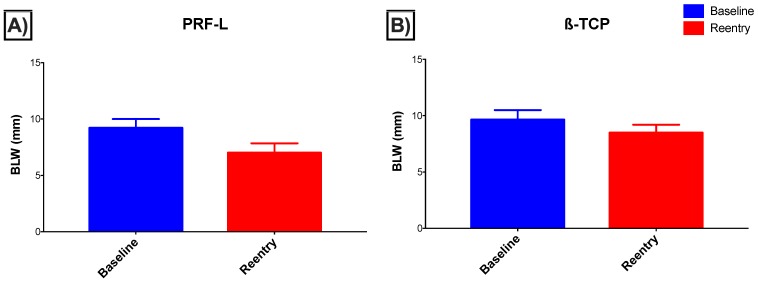
Bucco-lingual width (BLW) at baseline (blue) and reentry (red) (mean and standard deviation) for both (**A**) PRF-L and (**B**) β-TCP groups.

Finally, the bucco-lingual width (BLW) significantly changed from baseline to reentry in each group separately (*p* < 0.01, paired Student’s *t*-test) (Figure 2). Additionally, although no significant differences between groups were found at baseline, a wider alveolar crest was found in the β-TCP group at reentry (*p* < 0.01, independent Student’s *t*-test). Moreover, significantly more width was lost in the PRF-L group (*p* < 0.01, independent Student’s *t*-test).

**Figure 3:**
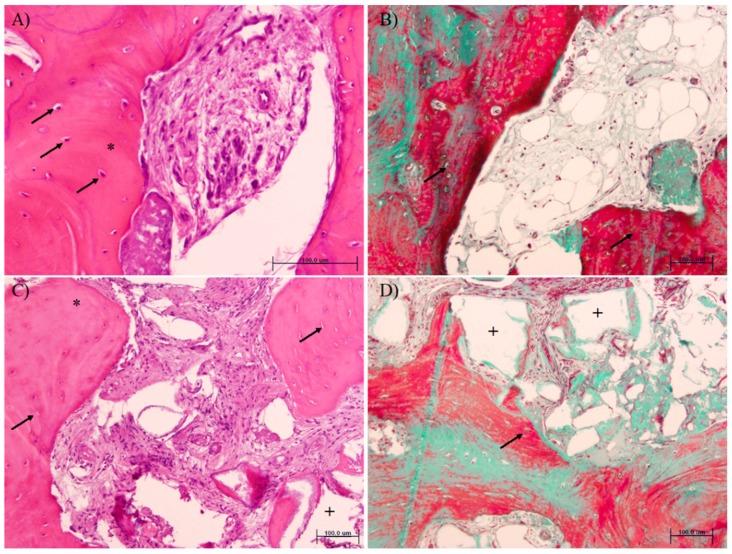
Histomorphometric analysis of samples from PRF-L (**A**,**B**) or β-TCP (**C**,**D**) treated sockets (hematoxylin and eosin (**A**,**C**) and Masson trichromic (**B**,**D**) stainings). Note that more osteocytes in the newly formed bone (arrows) and more new mineralized tissue (*) are present in the PRF-L group. Remnant particles (area left after demineralization, +) are only observed in the β-TCP group.

Histomorphometrically (Table 2 and Figure 3), we found more osteocytes in the newly formed bone after using PRF-L than when using β-TCP (123.25 (5.12) vs. 84.02 (26.53), respectively, *p* = 0.01, Mann–Whitney U test). Additionally, in the PRF-L group, more new mineralized tissue (77.33 (9.80) vs. 26.14 (7.49), *p* = 0.01, Mann–Whitney U test) but less non-mineralized tissue (22.67 (7.98) vs. 59.01 (2.23), *p* < 0.01, Mann–Whitney U test) were found. Obviously, no remaining particles were present in the PRF-L group.

## 4. Discussion

The aim of this study was to compare outcomes in ridge preservation using two different biomaterials after tooth extraction, and to analyze differences in the histological outcome of both clinical solutions. As has been previously well-defined [26,27,28], grafting does not preserve from the bone remodeling after extraction, and all biomaterials fail in the complete preservation of the initial alveolar bone, even after using different types of membranes [29]. Accordingly, in our study, both biomaterials led to a clinical resorption of the treated area (increase in ABC distance). Despite the reported advantage of PRF-L to promote soft tissue healing [30], in the current study β-TCP showed better results in terms of BLW and, particularly, in the MGJ final position. Conversely, a significantly higher percentage of new mineralized tissue could be observed in the samples from PRF-L grafted areas (77.33% (9.80) PRF-L vs. 26.14% (7.49) β-TCP). These observations can be based on different reasons. Firstly, in the PRF-L group, the potent osteoinductive effect that the proteins contained in platelet granules could have on wound healing may be the explanation for such observations. Gürbüzer et al. explained the effects of platelet concentrates on osteoblastic activity after four weeks of healing [31]. However, recently, Baslarli et al. failed to find significant differences between PRF-treated and non-PRF-treated sockets in terms of osteoblastic increased activity, claiming that PRF only has the potential characteristics of an autologous fibrin matrix that can accelerate the healing without those osteogenic effects [21]. Secondly, an augmented extension of soft tissue healing one week after tooth removal has also been proposed as an explanation [32,33]. The colour of the soft tissues, presence of bleeding on palpation, epithelialization of wound margins, granulation tissue and suppuration are additional characteristics of healing after use of PRF [34]. Thus, the quicker epithelization could lead to a better response in the hard tissue because of the protective factor of the keratinized mucosa and by the isolation of the wound from oral biofilm [35]. Thirdly, the presence of remnant particles of biomaterial, in the case of the β-TCP group, could promote some level of inflammatory response or delayed osteogenic response in the socket [36]. It is known that this family of biomaterials only shows osteoconductive properties, in contrast with the potent biological effect of growth factors present in the PRF-L concentrates, mainly transforming growth factor beta (TGF-β), platelet-derived growth factor (PDGF), vasculo-endothelial growth factor (VEGF), thrombospondin-1 (THBS-1), epidermal growth factor (EGF), interleukins and fibrin. Finally, the presence of such β-TCP particles could also limit the formation of new mineralized tissue by the direct effect of occupying the space and the biological effort used to resorb them. Other types of biomaterials have been regarded as being functionally incorporated into the trabecular structure, since the particles become recolonized by cells [37] and revascularized [38]. We have not found such properties for this β-TCP; thus, complete resorption is necessary to consider it functionalized and should not be accounted as part of the mineralized component of the bone.

A recent systematic review claimed that autologous platelet concentrates do not exert a noticeable effect on bone regeneration [35]. However, in the current study, we have found a high percentage of mineralized component in comparison to that obtained with other biomaterials. PRF-L induces higher osteoid areas [39]; thus, higher bone formation can ultimately be expected. In fact, our findings are related to those reported by Temmerman et al. [23], who found significant differences in socket filling after PRF-L (94.7%) versus natural healing (63.3%). However, data from Temmerman et al. must be interpreted with caution, because cone beam computed tomography (CBCT), although useful as a diagnostic tool, is an inadequate methodology to evaluate bone formation [40].

It is important to keep in mind that some grafts after four months of healing are expected to be completely resorbed, like the PRF-L, which in fact disappears even earlier. Thus, no biomaterial will remain in the sockets. Thus, the final quantity of mineralized and non-mineralized tissue will be influenced by the function and genetic pattern of each particular patient after the initial remodeling process. As a matter of fact, this event could explain the higher proportion of new formed mineralized tissue in this group, in an area classically classified as type I or II bone, highly mineralized. It is easy to understand that this biomaterial will promote a healing of the socket through osteoinduction, due to the proteins and growth factors. Other characteristics will also play a role, such as the size of the defect, location and patient features (age, gender, habits or systemic conditions) [41,42]. For example, sockets in the anterior mandible will show more new mineral structure than sockets in the posterior maxilla.

When using β-TCP, new mineralized bone was 26.14% (7.49) of the total grafted area while the remaining graft was 14.85% (6.40). These findings are similar to the proportions reported by Leventis et al. (24.4% (7.9) and 12.9% (7.7)) [43], but slightly inferior to other studies reported in the literature, like 47.7% (10.6) described by Mayer et al., although they used a combination of β-TCP and hydroxyapatite (HA) [44]. When HA grafts are used, new bone formation ranges from 1% [45] to 77.4% [46]. Other studies have found over 50% of new mineralized bone and less than 10% of residual graft particles when using bioactive glass [47,48]. It is important to point out that these differences might be related to a critical factor, namely, that their industrial production workflow and specific modifications, which vary the physico-chemical properties of the biomaterial and, accordingly, the biological response to them. Thus, different outcomes might be explained. In this sense, Monje et al. have demonstrated that allogenic biomaterials from the same source show differences in the features of the new tissue promoted, depending on whether they have been processed by solvent-dehydration or freeze-dried [49]. Other combinations of β-TCP have also been successfully tested and combined with purified stem cells [50]. Other examples include the modification of the biomaterial with poly (lactic-co-glycolic acid) (PLGA) coating that induces higher presence of undifferentiated cells and higher revascularization of the grafted area [51]. It seems obvious, then, that the differences observed between our results and those in the literature might be due to some of these factors.

In the β-TCP group, a collagen membrane was placed to prevent the release of biomaterial to the oral cavity and to favor the principles of guided bone regeneration [52]. Although the primary epithelization of the mucosa could be altered by the membrane, literature has provided evidence that histomorphometric results are similar, with an average of 45% and 42% of new bone and 36% and 43% of non-mineralized tissue in β-TCP + membrane and β-TCP alone, respectively [53]. In the PRF-L group, the collagen membrane was avoided because PRP concentrates alone show a barrier effect due to the cross-linked fibrin mesh [54].

In spite of the higher mineralized tissue in sockets filled with PRF-L, resorption was significantly higher than in the β-TCP filled sockets. It has been reported that although faster bone healing compared with the control group (natural healing) was observed, no statistically significant differences were detected [18]. In this sense, in our study PRF-L filled sockets showed a dimensional bucco-lingual width reduction of 2.19 (0.80) mm, going from the initial baseline of 9.19 (0.80) mm to 7.00 (0.85) mm after four months of healing. In contrast, β-TCP filled sockets showed a reduction of only 1.16 (0.55) mm, going from the initial baseline 9.64 (0.86) mm to 8.48 (0.71) mm, with statistically significant differences between groups and temporal frames (*p* < 0.001). Our data regarding dimensional changes were concordant with those explained in the literature. In a very similar study, although results obtained from PRF were almost similar to β-TCP combined with collagen (β-TCP-Cl), there were significant clinical and radiographic differences between the behavior of both biomaterials. The authors found less bucco-lingual resorption in the β-TCP-Cl group after six months: –3.85 mm in the PRF-L group vs. −3.15 mm in the β-TCP-Cl group (*p* < 0.001). Radiographically, the bucco-lingual dimensions changed by −1.53 mm in the PRF-L group (*p* < 0.001) vs. 0.33 mm for β-TCP-Cl (*p* = 0.031) [55]. However, although histological analysis was conducted in that study, histomorphometric data are not shown in the manuscript. Their histological images show a similar pattern to our findings, with more lamellar bone and a higher osteocyte density in the PRF-L group, suggesting an early maturation and remodeling of the bone in this group. This finding can be explained in two ways. On one side, in our study, 14.85% (6.40) of the space was occupied by remnant β-TCP particles, which may help to maintain the ridge volume. On the other hand, the healing could be accelerated in the sockets with PRF-L, thanks to the growth factors, which would lead to the quicker remodeling and maturation of the bone. This observation is based on the differences in the number of cells between both materials (osteocytes in newly formed bone per mm^2^ (123.25 (5.12) in PRF-L vs. 84.02 (26.53) in β-TCP, *p* = 0.01)).

In this study, we have correlated the clinical observations with the biological explanation. No radiographic data were used, which in fact could have helped to make these correlations useful in cases when no second surgery is needed. The raise of a full-thickness flap before tooth extraction may have also influenced the results related to MGJ final position. Despite these limitations, to our knowledge there are no previous studies that analyze the cellular components after using these biomaterials in socket preservation. As shown here, these factors should be studied as they may explain the clinical observations.

## 5. Conclusions

It can be concluded that after four months, PRF-L concentrate accelerates wound healing in post-extraction socket in terms of new mineralized bone component. However, the use of β-TCP biomaterial appears to be superior to maintain the bucco-lingual volume and the final position of mucogingival junction.

## Figures and Tables

**Table 1:** Demographic data by group and overall. PRF-L = platelet-rich fibrin group; β-TCP = beta tricalcium phosphate group.

	PRF-L *n* = 26	β-TCP *n* = 25
**Age** (mean(min–max))	47.72 (24–80) *	
	52.65 (24–80)	42.60 (25–66)
**Gender**	51 *	
Males	21 *	
	8	13
Females	30 *	
	18	12

* Overall data.

**Table 2:** Histomorphometric analysis of the samples taken from each group. PRF-L = platelet-rich fibrin group; β-TCP = beta tricalcium phosphate group.

	PRF-L	β-TCP	*p*-Value *
**Newly Formed Bone (Grafted Area)**			
Osteocytes (mm^2^)	123.25 (5.12)	84.02 (26.53)	0.01
Osteoblasts (mm^2^)	25.50 (1.29)	23.40 (2.63)	0.23
New mineralized tissue (%)	77.33 (9.80)	26.14 (7.49)	0.01
Non-mineralized tissue (%)	22.67 (3.98)	59.01 (2.23)	<0.001
Remnant graft (%)	0	14.85 (6.40)	0.01
**Native Bone**			
Osteocytes (mm^2^)	33.25 (2.50)	44.75 (4.01)	0.01
Osteoblasts (mm^2^)	9.75 (1.71)	25.80 (7.31)	0.01

* Mann–Whitney U test.
